# Stochastic three-dimensional numerical phantoms to enable computational studies in quantitative optoacoustic computed tomography of breast cancer

**DOI:** 10.1117/1.JBO.28.6.066002

**Published:** 2023-06-20

**Authors:** Seonyeong Park, Umberto Villa, Fu Li, Refik Mert Cam, Alexander A. Oraevsky, Mark A. Anastasio

**Affiliations:** a University of Illinois Urbana-Champaign, Department of Bioengineering, Urbana, Illinois, United States; b The University of Texas at Austin, Oden Institute for Computational Engineering and Sciences, Austin, Texas, United States; c University of Illinois Urbana-Champaign, Department of Electrical and Computer Engineering, Urbana, Illinois, United States; d TomoWave Laboratories, Houston, Texas, United States

**Keywords:** Numerical breast phantoms, optical breast phantoms, acoustic breast phantoms, optoacoustic computed tomography, photoacoustic computed tomography, virtual imaging trials

## Abstract

**Significance:**

When developing a new quantitative optoacoustic computed tomography (OAT) system for diagnostic imaging of breast cancer, objective assessments of various system designs through human trials are infeasible due to cost and ethical concerns. In prototype stages, however, different system designs can be cost-efficiently assessed via virtual imaging trials (VITs) employing ensembles of digital breast phantoms, i.e., numerical breast phantoms (NBPs), that convey clinically relevant variability in anatomy and optoacoustic tissue properties.

**Aim:**

The aim is to develop a framework for generating ensembles of realistic three-dimensional (3D) anatomical, functional, optical, and acoustic NBPs and numerical lesion phantoms (NLPs) for use in VITs of OAT applications in the diagnostic imaging of breast cancer.

**Approach:**

The generation of the anatomical NBPs was accomplished by extending existing NBPs developed by the U.S. Food and Drug Administration. As these were designed for use in mammography applications, substantial modifications were made to improve blood vasculature modeling for use in OAT. The NLPs were modeled to include viable tumor cells only or a combination of viable tumor cells, necrotic core, and peripheral angiogenesis region. Realistic optoacoustic tissue properties were stochastically assigned in the NBPs and NLPs.

**Results:**

To advance optoacoustic and optical imaging research, 84 datasets have been released; these consist of anatomical, functional, optical, and acoustic NBPs and the corresponding simulated multi-wavelength optical fluence, initial pressure, and OAT measurements. The generated NBPs were compared with clinical data with respect to the volume of breast blood vessels and spatially averaged effective optical attenuation. The usefulness of the proposed framework was demonstrated through a case study to investigate the impact of acoustic heterogeneity on OAT images of the breast.

**Conclusions:**

The proposed framework will enhance the authenticity of virtual OAT studies and can be widely employed for the investigation and development of advanced image reconstruction and machine learning-based methods, as well as the objective evaluation and optimization of the OAT system designs.

## 1 Introduction

Optoacoustic computed tomography (OAT), also known as photoacoustic computed tomography, is a non-invasive imaging modality being actively developed for clinical breast imaging and other biomedical applications.^1^^–^^8^ A unique feature of OAT is the ability to produce an image based on the endogenous optical contrast associated with chromophore concentrations and oxygenation states within tissue, without ionizing radiation and the loss of spatial resolution typically related to purely optical techniques such as diffuse optical tomography.^1^^,^^9^ This permits the imaging of tissue metabolism and angiogenesis, which have been identified to play a critical role in tumor growth and progression.^7^^,^^10^ Therefore, optoacoustic imaging is ideally positioned to resolve these two hallmarks of cancer *in vivo*.^2^^–^^8^^,^^10^ As such, an optimized and validated OAT system can be a powerful tool for the management of breast cancer. By assessing the tumor microvasculature density and blood oxygenation, it can enable the initial evaluation of tumor aggressiveness to inform the treatment plan and prognosis. It also allows for the monitoring of tumor response to treatment over time. However, to realize its full diagnostic potential, OAT should be equipped with the capability of providing quantitative information on true values of the optical absorption coefficient, which is proportional to molecular concentrations.^7^^,^^11^^,^^12^

A large number of different system designs for three-dimensional (3D) breast OAT that deploy varying light delivery and acoustic detection strategies have been proposed.^3^^–^^6^^,^^13^ This is unlike in X-ray mammography, breast magnetic resonance imaging (MRI), and breast ultrasound, in which similar implementations are typically in use per modality. Ideally, candidate designs of new quantitative OAT systems would be evaluated based on clinically relevant objective image quality measures via human subject studies. However, this is not a feasible solution given the large space of possible (technical trade-offs considered) design parameters of OAT imaging systems, the large variety in breast sizes and compositions, and the cost and potential ethical concerns associated with such studies. Instead of clinical trials, virtual imaging trials (VITs), i.e., computer-simulation studies, have been advocated for assessment and optimization of system and algorithm designs in the early stages of technology development. For VITs to be clinically relevant, realistic numerical phantoms must be employed as the to-be-imaged objects.^14^^–^^20^ Moreover and importantly, because imaging technologies are not typically optimized for use with only a single subject, ensembles of different phantoms that possess clinically relevant variability in anatomy and tissue properties must be virtually imaged. In this way, ensemble-averaged objective measures of image quality can be computed.^21^

Several numerical breast phantoms (NBPs) and numerical lesion phantoms (NLPs) have been proposed.^16^^–^^20^^,^^22^ However, most of the existing NBPs and NLPs were created from a limited number of clinical data, resulting in a lack of variability or oversimplified anatomical structures.^16^^–^^19^ The virtual imaging clinical trials for regulatory evaluation (VICTRE) project at the U.S. Food and Drug Administration (FDA)^14^ released software tools to generate realistic NBPs and NLPs for use in mammography applications. Although the VICTRE NBPs are considerably realistic for VITs of mammography in terms of principal tissue compositions, significant improvements are required for use in VITs of breast OAT. Bao et al.^20^ reported optoacoustic NBPs based on the VICTRE tools. However, these phantoms do not introduce adaptations of the blood vascular network for use in VITs of OAT and do not account for physiological variability in tissue optoacoustic properties, the values of which are fixed and deterministic for each tissue type.

Although simulation tools for photon transport and acoustic wave propagation^23^^–^^25^ in general media with spatially varying voxel-wise properties are available, the reported studies that employed NBPs did not leverage this capability. Not only in Ref. 20 but also in the other previous studies,^16^^–^^19^^,^^22^ piecewise-constant optical properties were assigned to each tissue type in the NBPs and NLPs without taking into account spatial variability in the property distribution induced by oxygen transport between tissues. In addition, a correlation between optical properties and both chromophore concentrations and the oxygenation state of the tissue has not been addressed in the existing NBPs and NLPs.^16^^–^^20^^,^^22^ Therefore, further enhancements are needed to establish physiologically realistic NBPs and NLPs for use in VITs of OAT.

In this work, an end-to-end framework for producing ensembles of realistic 3D anatomical, functional, optical, and acoustic NBPs and NLPs for use in VITs of quantitative OAT of breast cancer is established. The generation of the anatomical NBPs is accomplished by extending the VICTRE NBPs with blood vasculature modifications for use in VITs of OAT. Tissue composition within the anatomical NLPs is modeled according to different lesion types and aggressiveness. Depending on the type of cancer of interest, the lesions can contain a necrotic core and/or a tumor angiogenesis region surrounding the viable tumor cell region, which can be modeled because of the proposed modifications to the VICTRE NLPs. Realistic functional, optical, and acoustic properties are stochastically assigned for each breast tissue. The optical absorption coefficient is modeled based on the concentrations of the primary chromophores considered, which are stochastically chosen within their physiological ranges. Our modified version of the VICTRE tool code package,^26^ which is a fork of the original software, has been released under the same creative commons zero license (CC0) used by the original project. Furthermore, the software framework to generate stochastic distributions of the functional, optical, and acoustic properties based on the modified VICTRE anatomical phantoms has been made publicly available under the GNU general public license version 3 (GPLv3). The software, named stochastic optoacoustic NBP (SOA-NBP),^27^ is implemented in Python, and it includes capabilities for inserting superficial blood vasculature under the skin layer; creating anatomical NLPs; and assigning functional, optical, and acoustic properties to each breast tissue type. To enable researchers to immediately benefit from this work, 84 datasets were released under the CC0; these consist of anatomical, functional, optical, and acoustic NBPs and the corresponding simulated multi-wavelength optical fluence, initial pressure, and OAT measurement data.^28^^–^^30^

The remainder of this article is organized as follows. Relevant background information including a description of the VICTRE and previous NBPs for OAT is provided in Sec. 2. The proposed framework is described in Sec. 3, and several examples of NBPs generated employing the proposed framework are presented in Sec. 4. A case study that investigates the impact of acoustic breast heterogeneity on image reconstruction quality is provided in Sec. 5, as an example of how the proposed stochastic phantoms can enable important studies. The article concludes with a summary and discussion in Sec. 6.

## 2 Background

### 2.1 VICTRE Project by the FDA

The VICTRE project by the FDA^14^ developed software tools to generate 3D numerical representations of human female breasts and lesions for use in simulating X-ray mammography applications. These tools can create ensembles of stochastic, anatomically realistic breast structures and lesions within a user-defined 3D volume by specifying breast density (i.e., fat fraction), shape, size, and tissue composition parameters.^14^ The produced NBPs correspond to one of the following four types defined in the breast imaging reporting and data system (BI-RADS)^31^: (A) breast is almost entirely fatty, (B) breast has scattered areas of fibroglandular density, (C) breast is heterogeneously dense, and (D) breast is extremely dense. The tissue types in the VICTRE NBPs are fat, skin, glandular tissue, nipple, muscle, ligament, terminal duct lobular unit (TDLU), duct, artery, and vein.^14^ The VICTRE tools can also generate NLPs based on microcalcification clusters or spiculated masses.^14^ The NLPs are inserted at locations randomly selected from those predicted based on the duct and TDLU structures that are well-known sites for lesion formation.^32^

There exist several challenges that must be addressed to extend the VICTRE project to produce NBPs for use in VITs of breast OAT technologies. To perform such VITs, NBPs and NLPs that describe the optical and acoustic properties of the breast and lesion need to be established; they should be stochastic in nature and describe realistic values under typical physiological and pathological conditions for each tissue type. Because many 3D OAT technologies are not hand-held and utilize a fixed imaging geometry, the breast shape parameters need to be determined to be consistent with a prone position during a 3D OAT scan.^3^^–^^6^ Additionally, the representation of blood vasculature in the NBPs generated using the VICTRE tools, albeit sufficiently realistic for mammography applications,^14^ needs to be improved for realistic VITs of OAT because the VICTRE tool primarily produces deep-seated blood vessels rather than those near the skin layer that are dominantly exhibited in clinical OAT images.^3^^–^^6^^,^^33^ This is consequential because OAT has greater sensitivity to blood vessels than other tissues.^1^^,^^9^

### 2.2 Previous NBPs for OAT

Several NBPs for OAT have been proposed,^16^^–^^20^^,^^22^ and their features are summarized in Table 1. The NBPs in Refs. 17–19 have oversimplified structures, and only a handful of relatively thick blood vessels are included in the NBPs created via tissue segmentation from a limited number of X-ray mammography^22^ and MRI images.^16^ In Refs. 18, 19, and 22, a rounded-shaped NLP was inserted into the NBP, but the malignant tumors’ characteristics revealed in OAT images, such as tumor hypoxia and angiogenesis, were not modeled.

**Table 1:** Previous NBPs for OAT.

References	Phantom features
17	• 3D single breast tissue type and a cylindrical cyst
	• Optical absorption coefficient ($\mu_{a}$), reduced scattering coefficient, scattering anisotropy (g), and refractive index (n) for a wavelength of 1064 nm; sound speed (c) and acoustic attenuation coefficient ($\alpha_{0}$) with a power law exponent (y)
18 and 19	• 2D single breast tissue type and an elliptical^18^/circular^19^ tumor
	• $\mu_{a}$ and optical scattering coefficient ($\mu_{s}$) for a wavelength of 800 nm; c and density (ρ)^19^
16 ^a^	• 3D skin, fat, and fibroglandular tissues, as well as a handful of blood vessels, segmented from MRI images
	• $\mu_{a}$, $\mu_{s}$, g, and n for a wavelength of 760 nm; c and ρ
22	• 2D skin, fat, fibroglandular, and rounded tumor tissues, segmented from digital mammography
	• $\mu_{a}$ and $\mu_{s}$ for an unknown wavelength; c
20	• 3D NBPs of FDA’s VICTRE without any modifications for healthy breast tissues, and lesion models of fibroadenoma, DCIS, and IBC
	• $\mu_{a}$, $\mu_{s}$, g, and n for wavelengths of 700 and 900 nm; c and ρ

a Three datasets are publicly available.

Bao et al.^20^ presented FDA’s VICTRE NBPs with deterministic oxygen saturation and optical and acoustic properties assigned in a piecewise constant manner at two wavelengths of 700 and 900 nm. Also, the VICTRE NBPs were used without any modifications to the breast anatomy and shape, so the above mentioned challenges related to the blood vascular network and the patient’s position during the OAT scan remain unmet by this phantom design. The assignment of piecewise constant optical properties has been commonly adopted in most previous studies on numerical phantoms for OAT.^16^^–^^20^^,^^22^ However, this choice was mostly due to the need to reduce modeling and computational complexity. This limits the realism and usefulness of the generated optical phantoms. The optical absorption coefficient is determined by chromophore concentrations and hemoglobin oxygenation states within the tissue.^34^^,^^35^ Because the oxygen carried by blood diffuses through the surrounding tissue, the oxygen saturation distribution, and thus the optical absorption coefficient distribution, spatially varies in tissues. In Ref. 20, the oxygenation levels were neither considered nor correlated to the optical properties, except for the arteries and veins. The levels were set with fixed values of 95% for the arteries and 75% for the veins despite their varied values within a certain range.^36^ The optical properties at two wavelengths of 700 and 900 nm were directly assigned to the NBPs, and thus, these phantoms are unsuitable for VITs of breast multispectral OAT.

Oval-shaped fibroadenoma and irregularly shaped ductal carcinoma *in situ* (DCIS) and invasive breast cancer (IBC) were modeled in Ref. 20 using the VICTRE tool. As modifications to the VICTRE NLPs, a few thin veins were inserted inside the DCIS mass, and a centripetal vein and a surrounding arterial area with its irregularly shaped boundary were added for IBC. However, the characteristics and diameters of the inserted vasculature did not account for realistic lesion growth. Specifically, the features of the inserted vasculatures were based on clinical data of aggressive DCIS and IBC lesions larger than or equal to 47 mm in diameter,^37^ whereas the diameter of the simulated lesion was less than 10 mm. Furthermore, the method to generate the centripetal vein and the irregularly shaped arterial area was not explained in Ref. 20.

## 3 Methods

**Fig. 1:**
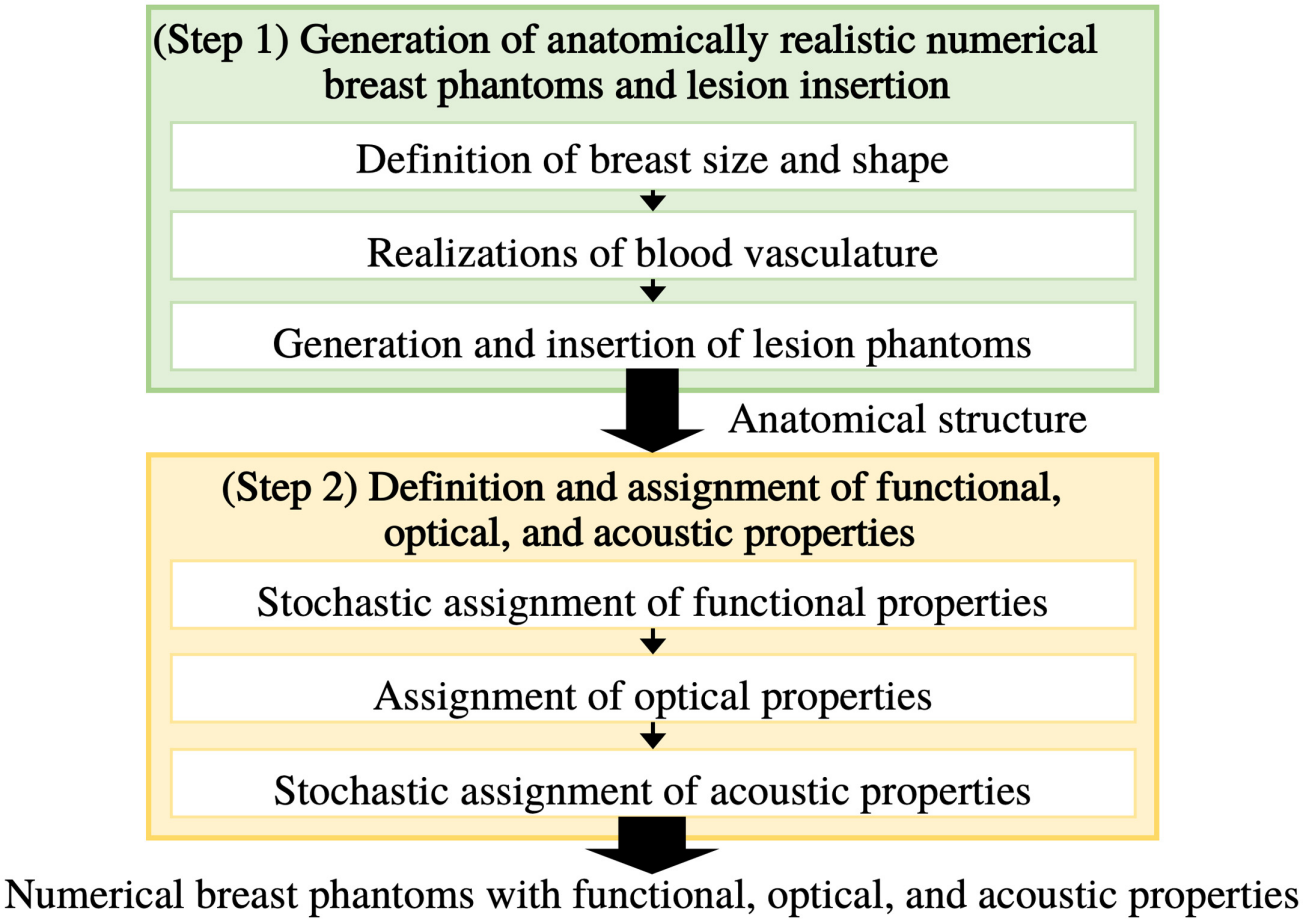
Generation of optoacoustic NBPs.

To circumvent the limitations described above, adaptations and customizations of the VICTRE NBPs were developed to enable the generation of large ensembles of realistic optoacoustic NBPs that exhibit clinically relevant variability in anatomical structures and functional, optical, and acoustic properties. The flow chart of optoacoustic NBP generation is illustrated in Fig. 1, and details of each step are explained in the following sections.

### 3.1 Generation of Realistic Anatomical NBPs and Lesion Insertion

In this first step, the construction of ensembles of realistic anatomical NBPs for different BI-RADS breast types [(A) breast is almost entirely fatty, (B) breast has scattered areas of fibroglandular density, (C) breast is heterogeneously dense, and (D) breast is extremely dense] using a modified version of the VICTRE tool is described. Additionally, the generation and insertion of anatomical NLPs into the NBPs are described.

#### 3.1.1 Definition of breast size and shape

Based on clinical data and constraints by designs of the existing OAT breast imaging systems^3^^–^^6^ (where a breast should fit within a scanning radius of 85 mm), the distributions of breast size parameters were determined for each BI-RADS breast type.^38^ The VICTRE tool generates anatomical NBPs according to the parameters specified in the configuration file. Among the parameters, the breast volume extent parameters $a_{1t}$, $a_{1b}$, $a_{2l}$, $a_{2r}$, and $a_{3}$ and the breast shape parameters $\epsilon_{1}$, $B_{0}$, $B_{1}$, $H_{0}$, and $H_{1}$ were specified in the VICTRE configuration file. These parameters are explained in Ref. 15, and the probability distributions for the parameters that are consistent with the patient’s position during a 3D OAT scan are summarized in Table 2. Here, a truncated Gaussian distribution (TN) was chosen among the possible distributions because it is the maximum entropy distribution supported in a bounded interval with a specified mean and standard deviation.^39^

**Table 2:** Shape and size parameters.

Parameter	Types A and B	Type C	Type D
$a_{1t}$ (mm)	TN(59.70,3.58,50.77,71.5)		TN(50.05,3.58,42.9,57.2)
$a_{1b}/a_{1t}$	N(1,0.02)		
$a_{2r}/a_{1t}$	N(1,0.05)		
$a_{2l}/a_{2r}$	N(1,0.05)		
$a_{3}/a_{1t}$	TN(0.85,0.14,0.8,1.2)	TN(0.85,0.12,0.7,1.1)	TN(0.85,0.1,0.7,1.1)
$\epsilon_{1}$	N(1,0.1)		
$B_{0}$	TN(0,0.1,−0.18,0.18)		
$B_{1}$	TN(0,0.1,−0.18,0.18)		
$H_{0}$	TN(0,0.15,−0.11,0.11)		
$H_{1}$	TN(0,0.25,−0.3,0.3)		

N(μ,σ): Gaussian distribution with mean μ and standard deviation σ.

TN(μ,σ,a,b): truncated Gaussian distribution in interval (a,b).

For hemispherical shapes ($a_{1t}=a_{1b}=a_{2r}=a_{2l}=a_{3}$), the $\epsilon_{1}$ value is set to “1,” and the $B_{0}$, $B_{1}$, $H_{0}$, and $H_{1}$ values are set to “0.”

The generated anatomical NBPs were discretized in three dimensions using a uniform Cartesian lattice. A voxel size of 0.125 mm was chosen for the discretization, considering computation time and memory usage. This choice allows for avoiding the discretization inverse crime when performing image reconstruction as the typical resolution in OAT breast images is between 0.2 and 0.5 mm. Each voxel value corresponds to an unsigned 8-bit integer tissue label.^14^ The anatomical NBPs were rotated to be consistent with a prone position during a 3D OAT scan and cropped to exclude the chest muscle region.

#### 3.1.2 Realizations of blood vasculature

**Fig. 2:**
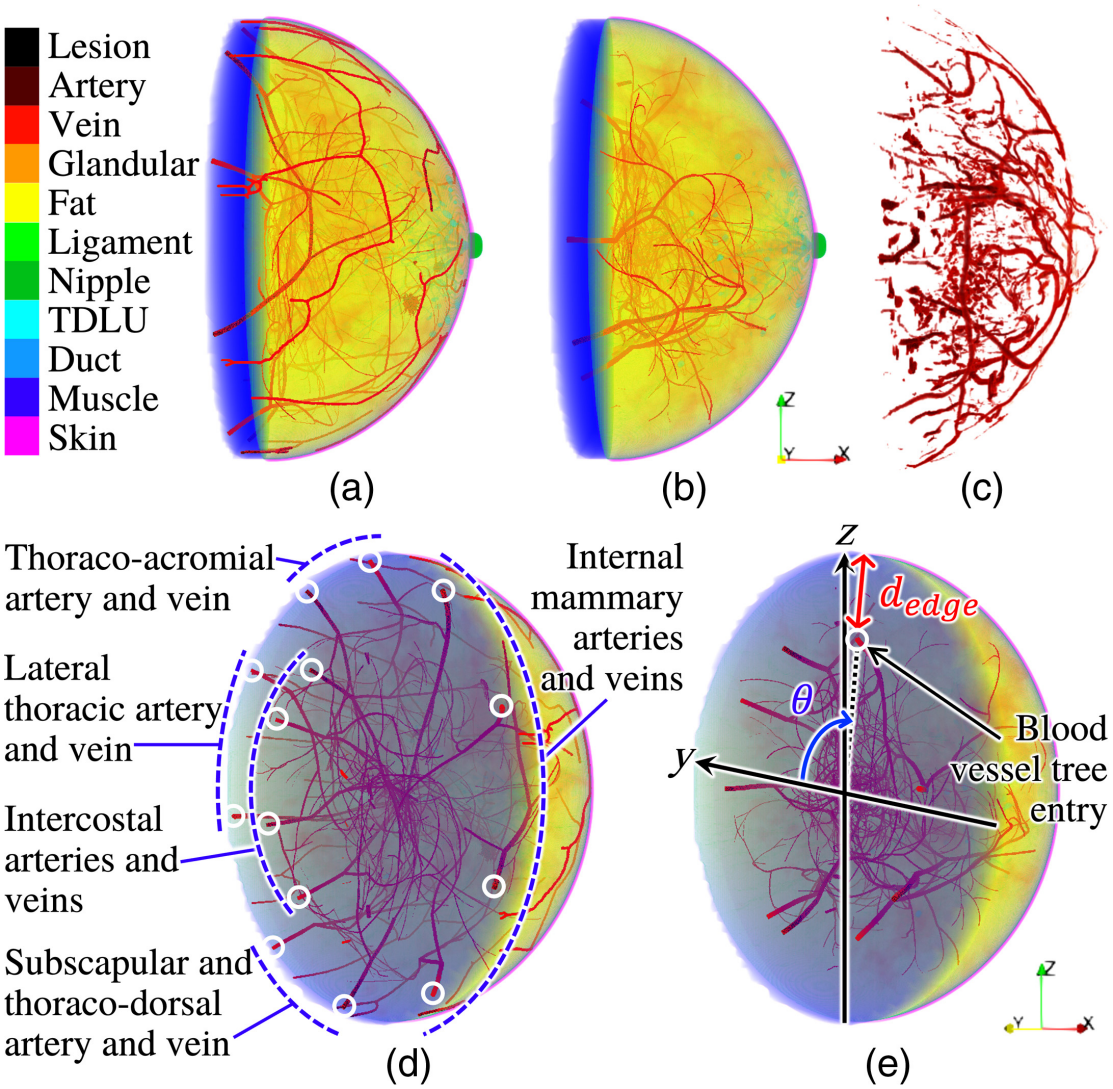
Blood vessels in an NBP (type B, left breast) with (a and d) and without (b and e) blood vasculature customization and (c) a clinical OAT image acquired by TomoWave Laboratories employing LOUISA-3D^3^ at the MD Anderson Cancer Center and postprocessed to extract blood vascular structures.^33^ Paraview^40^ was used for volume rendering.

A specific innovation in our adaptation is the introduction of realistic blood vasculature, as shown in Figs. 2(a) and 2(d). The VICTRE tool iteratively generates sibling and child branches of arterial and venous blood vessel trees within the breast volume, using randomly sampled values based on blood vessel parameters (Table 4) in the configuration file.^20^ However, the tool assumes four arterial and five venous blood vessel trees with their entry locations predefined by fixed polar angles θ and a fixed distance from the breast edge $d_{\text{edge}}=20\;\mathrm{mm}$ [Fig. 2(e)]. This is inconsistent with the anatomy of the breast. Furthermore, the values were hardcoded within the C++ source code of the VICTRE tool, making it inaccessible for users to adjust them through the configuration file. Thus, to more realistically model breast anatomy,^41^ the source code of the VICTRE tool was modified to incorporate five sets of blood vessel trees: internal mammary, thoraco-acromial, lateral thoracic, subscapular and thoraco-dorsal, and intercostal arteries and veins [Fig. 2(d)]. Two tunable parameters, vesselEdgeSep1 and vesselEdgeSep2 [$d_{\text{edge}}$ in Table 3], were added to the configuration file to set the distances from the breast edge (1) to the internal mammary, thoraco-acromial, lateral thoracic, and subscapular and thoraco-dorsal arteries and veins and (2) to the intercostal arteries and veins, respectively [Fig. 2(d)]. Table 3 summarizes the entry locations of the blood vessel trees in the left breast, and those in the right breast were set to be bilaterally symmetric.

**Table 3:** Entry locations of blood vessel trees.

Blood vessel	θ (rad), artery	θ (rad), vein	$d_{\text{edge}}$ (mm)
Internal mammary	11π/15, −8π/9	41π/45, −32π/45	2
Thoraco-acromial	2π/5	17π/30	
Lateral thoracic	−2π/45	π/5	
Subscapular and thoraco-dorsal	−π/2	−13π/45	
Intercostal	16π/45, −π/12	π/6, −3π/10	24

θ: angle in the polar coordinate system.

$d_{\text{edge}}$: distance from the edge of the breast along the radial direction.

Among blood vessel tree, branch, and segment parameters in the VICTRE tool, the parameters maxBranch, initRad, minRadFrac, numTry, maxTry, and absMaxTry provided in Table 4 were tuned based on the breast volume percentage occupied by blood vessels estimated from four clinical OAT datasets. The clinical datasets used as references were acquired by TomoWave Laboratories (Houston, Texas, United States) using LOUISA-3D^3^ at the MD Anderson Cancer Center. The experimental 3D OAT images were reconstructed using a filtered back-projection (FBP) method.^42^ To compute the breast volume percentages occupied by blood vessels from the reconstructed images, the distributions of the total hemoglobin concentration were estimated by employing spectral linear unmixing with optical fluence normalization.^33^ Then, a vessel enhancement filter^43^ was applied to the estimated distributions of the total hemoglobin concentration [Fig. 2(c)], and the ratio of the numbers of the blood vessel voxels and breast voxels was calculated.

**Table 4:** Blood vessel parameters.

Parameter	Description	Value
maxBranch	Target number of branches	500
initRad	Initial radius of tree (mm)	0.6
minRadFrac	Minimum starting radius as a fraction of parent end radius	0.85
numTry	Number of trial segments to generate	50
maxTry	Maximum number of segments to generate before reducing length	100
absMaxTry	Total number of segment tries	1000

The blood vessels within the depth of 10 mm under the skin layer are dominantly observed with relatively high image contrast in clinical OAT images, i.e., estimated distributions of the initial pressure, but are missing in the VICTRE NBPs [Figs. 2(b) and 2(e)]. To address this, additional blood vasculature [Figs. 2(a) and 2(d)] was stochastically generated using computationally efficient methods (random sampling, Gaussian blurring, Otsu’s thresholding,^44^ and skeletonization), which are commonly used in image processing. The diameter of the blood vessels and their depth from the skin are tunable and were set to 0.75 and 0.375 mm, respectively. Multiple blood vessel segments were formed and alternately divided into arteries and veins depending on the segment locations. Instead of the proposed method, another user-defined method can be employed in our proposed framework if desired. Details of the blood vasculature generation are provided in Appendix A.

#### 3.1.3 Generation and insertion of lesions

**Fig. 3:**
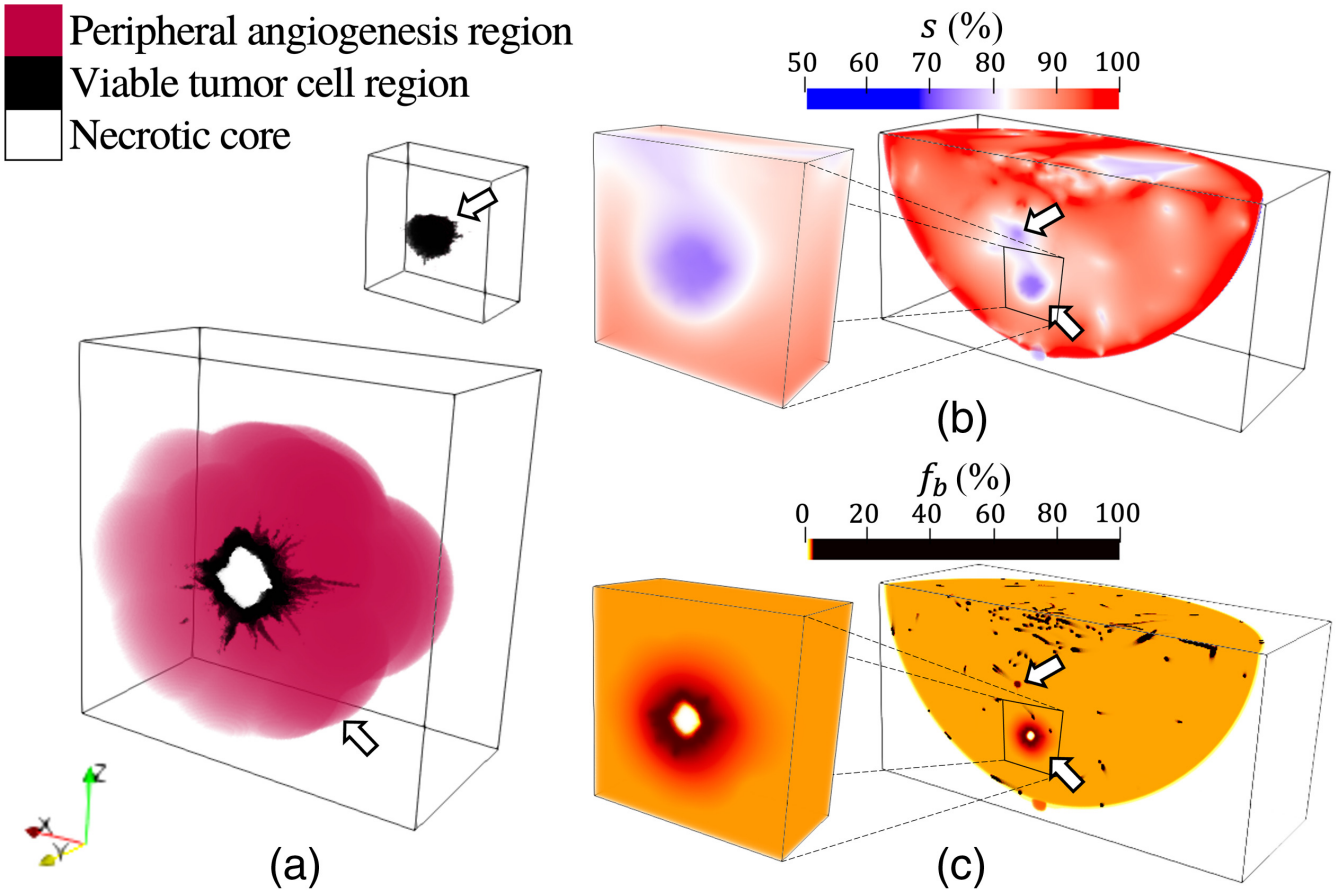
Malignant lesion model: (a) anatomical NLPs without (top) and with a necrotic core and a peripheral angiogenesis region (bottom), and distributions of (b) oxygen saturation s and (c) blood volume fraction $f_{b}$. The two lesions were inserted at physiologically plausible locations randomly selected among the candidate sites produced by the VICTRE tools. In panels (a)–(c), halves of the lesion volumes are presented to show their cross-sections. In panels (b) and (c), the partial breast volumes clipped at the y-coordinate at which both lesions are exhibited are illustrated. The arrows in panel (b) indicate the simulated tumor hypoxia and those in panel (c) indicate the simulated tumor angiogenesis, necrotic tumor core, and relatively high total hemoglobin concentration of the viable tumor cells compared with healthy tissues. These are from a type A breast. Paraview^40^ was used for volume rendering, and color maps were adjusted for better visibility.

Most common types of malignant lesions, such as invasive ductal carcinoma and invasive lobular carcinoma, have anatomically irregular outlines and develop in milk ducts and lobules.^32^ Depending on the type of lesion and its aggressiveness, the lesion growth can be accompanied by severe hypoxia in the center region, tumor necrosis, and tumor angiogenesis in the periphery of the viable tumor cells.^45^^–^^47^ Users can model lesions as being composed solely of a viable tumor cell (VTC) region or containing a necrotic core, VTC region, and peripheral angiogenesis (PA) region [Fig. 3(a)].^47^^–^^49^ The irregular spiculated boundary of the VTC region was created employing the VICTRE tool. The necrotic core region and PA region were formed via erosion and dilation operations^50^ applied to the surface of the VTC region using spherical structuring elements, i.e., matrices that identify the voxel and its neighboring voxels within a spherical area to be eroded or dilated, respectively. Here, the radii of the spherical structuring elements were 0.75 mm (the thickness of the ring-shaped VTC region)^49^ for the erosion and 5 mm^48^ for the dilation. The generated anatomical NLPs can be optionally inserted into the anatomical NBP. Among the candidate lesion locations predicted via the VICTRE tools, those that do not overlap with other (already inserted) lesions or the skin, nipple, and muscle were randomly chosen as the sites at which to insert the NLP.

### 3.2 Definition and Assignment of Functional, Optical, and Acoustic Properties

Functional, optical, and acoustic NBPs can be separately established via assignment of the specific properties of breasts to each tissue type in the anatomical NBPs described in Sec. 3.1. The optical contrast exhibited in OAT images is determined by light absorption by chromophores, and the concentration distributions and molar extinction coefficients of the chromophores determine the optical absorption distribution in the tissue.^34^ The primary chromophores of the breast relevant to OAT are oxy- and deoxy-hemoglobin, water, fat, and melanin. The hemoglobin concentration in the tissue can be described by the total hemoglobin concentration in blood and the volume fraction of blood in the tissue, whereas the melanin concentration in the epidermis can be assumed to be proportional to the volume fraction of melanosome, an organelle where melanin is synthesized.^34^ The volume fraction of the optical absorption coefficient of a pure chromophore can be a surrogate of its concentration in the tissue.^34^^,^^51^^–^^54^ In the proposed method, therefore, such functional properties were assigned to each tissue type in the anatomical NBPs, and then the corresponding optical properties were computed and assigned. Sections 3.2.1–3.2.3 elaborate on how the functional, optical, and acoustic properties were stochastically assigned to each tissue type. The prescribed statistical characteristics of the functional, optical, and acoustic properties were informed by a comprehensive literature survey to faithfully represent anatomically and physiologically realistic values and variations.^15^^,^^34^^–^^36^^,^^51^^–^^80^

#### 3.2.1 Stochastic assignment of functional properties

NBPs that describe the functional properties of the breast tissues without and with lesions were established as follows. The functional properties considered were the total hemoglobin concentration of blood $c_{\mathrm{tHb},\text{blood}}$ (μM); oxygen saturation s (%); and volume fractions of blood $f_{b}$, water $f_{w}$, fat $f_{f}$, and melanosome $f_{m}$ (%). Although other chromophores exist, their contribution to the optical absorption coefficient at the wavelength in the near infrared (NIR) range (700 to 1100 nm) is negligible, so they are omitted. For this reason, the sum of the volume fractions of the primary chromophores ($f_{b}+f_{w}+f_{f}+f_{m}$) in Table 5 is always less than or equal to 100%. The value of $c_{\mathrm{tHb},\text{blood}}$ for each NBP was randomly sampled from a uniform distribution U(1860,2325)  μM corresponding to a normal hematocrit level (36% to 44%) for women.^55^

**Table 5:** Functional properties of breast tissues and lesion.

Medium	s (%)	$f_{b}$ (%)	$f_{w}$ (%)	$f_{f}$ (%)	$f_{m}$ [%]
Fat/ligament/TDLU/duct^51^	PDE^a^	TN(1.15,0.22,0.91,1.43)	TN(29.17,13.11,14,40)	$100-(f_{b,\mathrm{fat}}+f_{w,\mathrm{fat}})$	0
Glandular^51^	PDE^a^	$f_{b,\text{fat}}$	$f_{w,\text{fat}}$	0	0
Skin^52^	98.9	0.39	TN(18.68,1.34,12,25)	TN(30.72,3.79,12,48)	TN(0.64,0.04,0.44,0.84)
Nipple^53^	71.28	1.35	TN(45.4,11.7,25.1,76.6)	$100-(f_{b,\text{nipple}}+f_{w,\text{nipple}}+f_{m,\text{nipple}})$	TN(0.82,0.09,0.4,1.24) ^52^
Artery	U(95,99) ^36^	100	0	0	0
Vein	U(75,84) ^36^	$f_{b,\text{artery}}$	0	0	0
VTC^54^	TN(69.91,4.99,62.5,76.49)	TN(1.64,0.6,0.89,2.93)	TN(47.67,20.15,24.14,82.25)	$100-(f_{b,\mathrm{VTC}}+f_{w,\mathrm{VTC}})$	0
Necrotic core^54^	$s_{\mathrm{VTC}}$	0	$f_{w,\mathrm{VTC}}$	$f_{f,\mathrm{VTC}}$	0
PA^54^	PDE^a^	PDE^a^	$f_{w,\mathrm{VTC}}$	$f_{f,\mathrm{VTC}}$	0

a The PDE formulation creates a smooth transition for the tissue property distribution.

##### NBPs without lesions

Functional property values (s, $f_{b}$, $f_{w}$, $f_{f}$, and $f_{m}$) for each breast tissue type were sampled from the predefined probability distributions and assigned to the corresponding tissue voxels in the anatomical NBPs [Sec. 3.1]. A comprehensive literature survey was conducted to faithfully represent physiologically realistic values and variations,^36^^,^^51^^–^^54^ and Table 5 provides the probability distributions of the functional properties defined for each tissue type. To mimic physiological spatial variation in the oxygen saturation distribution, the oxygen saturation in the tissues was modeled by solving a diffusion-reaction partial differential equation (PDE)^81^ using FEniCS,^82^ which is an open-source finite element library for solving PDEs. Specifically, the oxygen saturation s values sampled from the probability distributions in Table 5 were assigned to the specific tissues (skin, nipple, artery, and vein). Then the solution to the PDE was used to define the s distribution in the surrounding tissues (fat, ligament, TDLU, duct, and glandular tissues).

##### NBPs with lesions

For the NBPs containing malignant lesions, tumor hypoxia^11^ was mimicked by assigning a relatively low s value sampled from the probability distribution in Table 5 to the voxels corresponding to the VTC region and necrotic core in the anatomical NBP [Fig. 3(b)]. More details are provided in Appendix B. In addition to tumor hypoxia, other features of aggressive malignant lesions are (1) a relatively high total hemoglobin concentration, i.e., blood volume fraction $f_{b}$, in the lesion and its peripheral region (tumor angiogenesis) compared with healthy tissues and (2) tumor necrosis.^45^^–^^47^ For aggressive malignant lesions, the $f_{b}$ distribution was modeled to mimic tumor necrosis by assigning an $f_{b}$ value of 0% to the necrotic core. The capillaries newly sprouted from the vascularized lesions have diameters ranging from 3 to 40  μm^45^ and, therefore, are too small to be geometrically resolved in the discretized NBP (a voxel size of 0.125 mm). For this reason, the presence of such capillaries was accounted for by assigning a spatially varying local increase in blood volume fraction $f_{b}$ within the VTC and PA regions [Fig. 3(c)]. Additional details are presented in Appendix B.

#### 3.2.2 Assignment of optical properties

NBPs that describe the optical properties of the breast tissues were established as follows. The optical properties considered were optical absorption coefficient $\mu_{a}$ (${\mathrm{mm}}^{-1}$), optical scattering coefficient $\mu_{s}$ (${\mathrm{mm}}^{-1}$), scattering anisotropy g, and refractive index n. Illumination wavelengths were selected from the NIR spectral range from 700 to 1100 nm, which is commonly used in OAT breast imaging.^3^^–^^6^^,^^13^ The optical properties are wavelength-dependent; however, g and n do not vary significantly over the NIR range.^34^ Thus, constant g and n values were defined for each tissue type regardless of the wavelength and were assigned to the corresponding tissue voxels, as presented in Table 6. For the PA region, the g and n values of its underlying tissues (fat/ligament/TDLU/duct or glandular tissues) were assigned to the corresponding voxels.

**Table 6:** Scattering coefficient parameters, scattering anisotropy, and refractive index of breast tissues and lesion.

Medium	${\mu_{s}}^{\prime }(\lambda_{\mathrm{ref}})$ (${\mathrm{mm}}^{-1}$)	b	g	n
Fat/ligament/TDLU/duct	0.83^61^	0.617^61^	0.98^62^	1.44^63^
Glandular	1.06^64^	0.52^64^	0.96^62^	1.36^63^
Skin/nipple	(3.72, 4.78)^65^^,^^66^	(1.39, 2.453)^65^^,^^66^	0.65^67^	1.37^68^
Artery/vein	(2.2, 2.295)^69^^,^^70^	(0.66, 0.872)^69^^,^^70^	0.976^71^	1.35^72^
VTC/necrotic core	(2, 2.07)^61^^,^^62^	(0.725, 1.487)^61^^,^^62^	0.955^62^	1.39^73^

A reference wavelength ($\lambda_{\mathrm{ref}}$) is 500 nm.

The $\mu_{a}$ value at $r=(x,y,z)\in R^{3}$ at a wavelength of λ was calculated based on the specified functional NBPs as^34^^,^^35^

$$\mu_{a}(r,\lambda )=\underset{i\in I}{\sum }f_{i}(r)\mu_{a,i}(\lambda ),$$ (1)

where I={ oxygenated blood, deoxygenated blood, water, fat, melanosome } is a set of chromophores of interest. $f_{i}(r)$ and $\mu_{a,i}(\lambda )$ are the volume fraction at r and the optical absorption coefficient at a wavelength of λ of the pure chromophore indexed by i, respectively. The volume fractions of oxygenated blood $f_{\text{oxyblood}}(r)=f_{b}(r)s(r)$ and deoxygenated blood $f_{\text{deoxyblood}}(r)=f_{b}(r)\{1-s(r)\}$ were computed using the blood volume fraction $f_{b}(r)$ and oxygen saturation s(r) defined in Sec. 3.2.1. The corresponding optical absorption coefficients were computed as $\mu_{a,\text{oxyblood}}(\lambda )=\ln(10)c_{\mathrm{tHb},\text{blood}}\epsilon_{{\mathrm{HbO}}_{2}}(\lambda )$ for oxygenated blood and $\mu_{a,\text{deoxyblood}}(\lambda )=\ln(10)c_{\mathrm{tHb},\text{blood}}\epsilon_{\mathrm{Hb}}(\lambda )$ for deoxygenated blood, where $\epsilon_{{\mathrm{HbO}}_{2}}$ and $\epsilon_{\mathrm{Hb}}$ are molar extinction coefficients (${\mathrm{mm}}^{-1}\;M^{-1}$) of oxy- and deoxy-hemoglobin, respectively. The values of $\epsilon_{{\mathrm{HbO}}_{2}}$, $\epsilon_{\mathrm{Hb}}$, and optical absorption coefficients of water, fat, and melanosome for the NIR range were from the data in Refs. 35 and 57–59.

The $\mu_{s}$ value was calculated according to the power law^34^ as

$$\mu_{s}(r,\lambda )=\frac{{\mu_{s}}^{\prime }(r,\lambda )}{1-g(r)}=\frac{{\mu_{s}}^{\prime }(r,\lambda_{\mathrm{ref}})}{1-g(r)}{\left(\frac{\lambda }{\lambda_{\mathrm{ref}}}\right)}^{-b(r)},$$ (2)

where $\lambda_{\mathrm{ref}}$ is a reference wavelength of 500 nm and ${\mu_{s}}^{\prime }(r,\lambda_{\mathrm{ref}})$ and b(r) are a reduced scattering coefficient (${\mathrm{mm}}^{-1}$) at a wavelength of $\lambda_{\mathrm{ref}}$ and a scattering power law exponent, respectively. To account for the correlation between ${\mu_{s}}^{\prime }(r,\lambda_{\mathrm{ref}})$ and b(r) observed from data in Ref. 34, both values were jointly sampled as

$${\mu_{s}}^{\prime }(r,\lambda_{\mathrm{ref}})=[{\mu_{s,u}}^{\prime }(r,\lambda_{\mathrm{ref}})-{\mu_{s,l}}^{\prime }(r,\lambda_{\mathrm{ref}})]X+{\mu_{s,l}}^{\prime }(r,\lambda_{\mathrm{ref}})\;\text{and}\;b(r)=[b_{u}(r)-b_{l}(r)]X+b_{l}(r),$$ (3)

where X∼U(0,1) is a random variable, ${\mu_{s,u}}^{\prime }(\lambda_{\mathrm{ref}})$ and ${\mu_{s,l}}^{\prime }(\lambda_{\mathrm{ref}})$ correspond to upper and lower bounds of ${\mu_{s}}^{\prime }(\lambda_{\mathrm{ref}})$, respectively, and $b_{u}$ and $b_{l}$ are upper and lower bounds of b, respectively. These values for each tissue type are summarized in Table 6. The $\mu_{s}$ value of water at a wavelength of λ was assigned with an estimate from a curve based on Eq. (2) that fits to the measurements reported in Ref. 60. The VTC and necrotic core regions have a relatively high $\mu_{s}$ value due to tumor cell proliferation, compared with healthy tissues^83^ [see Table 6]. For the PA region in the NBP, the $\mu_{s}$ values of the underlying tissues (fat/ligament/TDLU/duct or glandular tissues) were assigned to the corresponding voxels.

#### 3.2.3 Stochastic assignment of acoustic properties

Acoustic NBPs for ultrasound computed tomography (USCT) were proposed by some of the authors in Ref. 15, with the tissues that are invisible in USCT imaging being excluded from consideration. For virtual OAT imaging, NBPs that describe the acoustic properties of the breast tissues were established as follows. The acoustic properties considered were sound speed c (mm/μs), density ρ (${g/\mathrm{mm}}^{3}$), and acoustic attenuation coefficient $\alpha_{0}$ (${\mathrm{dB}/\mathrm{MHz}}^{y}\;\mathrm{mm}$) with power law exponent y. Table 7 provides the probability distributions of c, ρ, and $\alpha_{0}$ for each tissue type.^15^ For the PA region, similar to the assignment of $\mu_{s}$, g, and n values in Sec. 3.2.2, the acoustic properties of the underlying tissues (fat/ligament/TDLU/duct or glandular tissues) were assigned to the corresponding voxels. Several widely used time-domain wave propagation simulators assume a spatially homogeneous y^25^ although the exponent y varies between 1 and 1.5 depending on the tissue type.^84^ Accordingly, the homogeneous values of 1.1151, 1.1642, 1.2563, and 1.3635 were used for breast types A to D, respectively, as reported in Ref. 15. Once the simulators start supporting spatially varying y distributions and tissue type-dependent y data becomes available, it will be possible to investigate the error induced by the spatially homogeneous y.

**Table 7:** Acoustic properties of breast tissues and lesion.

Medium	c (mm/μs)	ρ (${g/\mathrm{mm}}^{3}$)	$\alpha_{0}$ (${\mathrm{dB}/\mathrm{MHz}}^{y}\;\mathrm{mm}$)
Water^a^	1.521^74^	$0.993\times 10^{-3}$ ^75^	$2.2\times 10^{-4}$ ^75^
Fat	TN(1.44,0.021,1.41,1.49) ^76^ ^,^ ^77^	$\mathrm{TN}(0.911,0.053,0.812,0.961)\times 10^{-3}$ ^75^	N(0.038,0.004) ^75^
Glandular/ TDLU/duct	TN(1.54,0.015,1.517,1.567) ^76^ ^,^ ^77^	$\mathrm{TN}(1.041,0.045,0.99,1.092)\times 10^{-3}$ ^75^	N(0.075,0.008) ^75^
Ligament	TN(1.457,0.019,1.422,1.496) ^76^ ^,^ ^77^	$\mathrm{TN}(1.142,0.045,1.1,1.174)\times 10^{-3}$ ^75^	N(0.126,0.013) ^75^
Skin/nipple	TN(1.555,0.01,1.53,1.58) ^76^	$\mathrm{TN}(1.109,0.014,1.1,1.125)\times 10^{-3}$ ^75^	N(0.184,0.019) ^75^
Artery/vein	TN(1.578,0.011,1.559,1.59) ^75^	$\mathrm{TN}(1.05,0.017,1.025,1.06)\times 10^{-3}$ ^75^	0.021^75^
VTC/necrotic core	TN(1.548,0.01,1.531,1.565) ^78^	$\mathrm{TN}(0.945,0.02,0.911,0.999)\times 10^{-3}$ ^79^	N(0.269,0.02) ^80^

a Acoustic properties of water are consistent with an assumed temperature of 37°C, which is often used in breast OAT to minimize patient discomfort.

## 4 Examples of Generated NBPs and Corresponding OAT Images

To provide insight into the visual and quantitative characteristics of NBPs created by the proposed framework and the corresponding OAT images, example NBPs were produced as explained in Sec. 4.1. Subsequently, OAT measurement data were simulated based on a target imaging system described in Sec. 4.2, and 3D estimates of the induced pressure distributions were reconstructed using an FBP method.

### 4.1 Examples of Generated NBPs

**Fig. 4:**
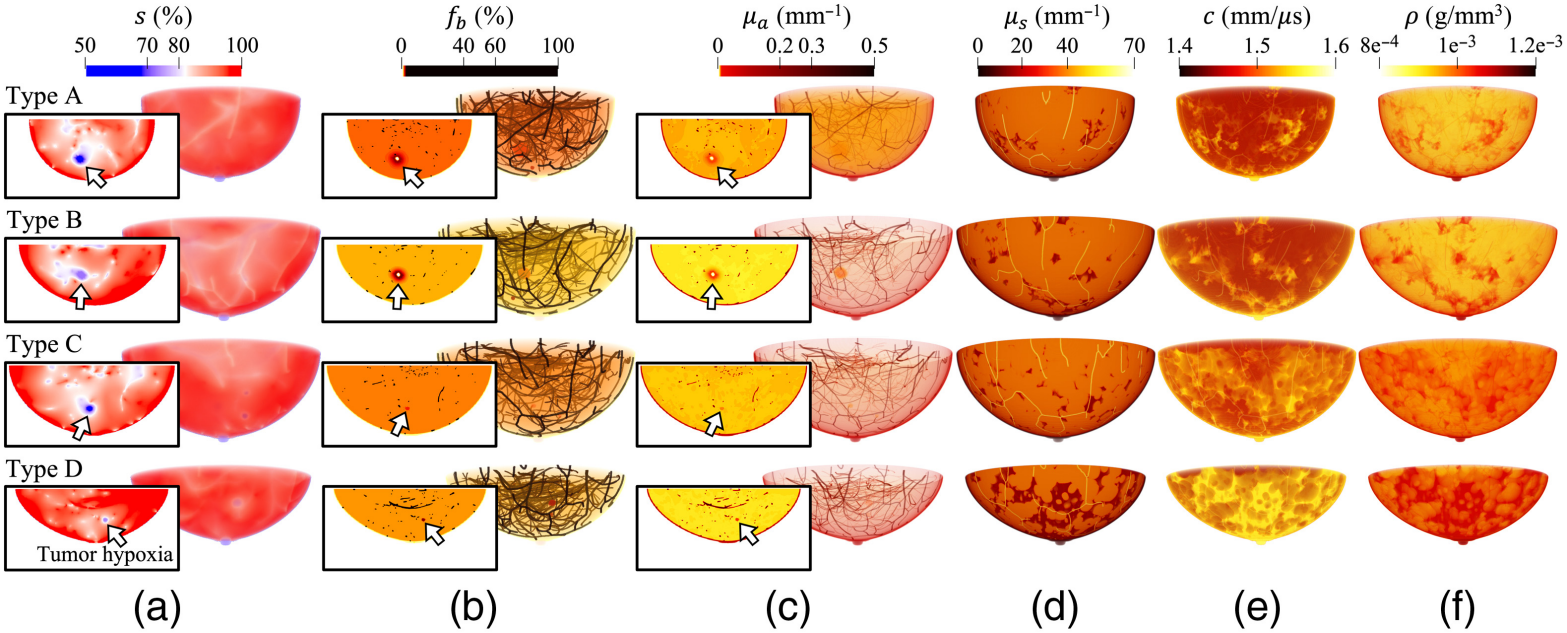
Distributions of functional, optical, and acoustic properties: (a) oxygen saturation s, (b) blood volume fraction $f_{b}$, (c) optical absorption coefficients $\mu_{a}$, and (d) optical scattering coefficients $\mu_{s}$ at a wavelength of 800 nm, (e) sound speed c, and (f) density ρ of type A to D breasts (top to bottom). The type A and B breasts (top two rows) have two lesions inserted, one with a necrotic core and PA region and the other without, whereas the type C and D breasts (bottom two rows) have four lesions of different sizes inserted, each smaller than 10 mm in diameter and composed solely of a VTC region. Insets of (a)–(c) show cross-sections, with the lesion location indicated with arrows. Paraview^40^ was used for volume rendering, and color maps were adjusted for better visibility.

A total of 84 NBPs of various sizes, 21 for each breast type [(A) breast is almost entirely fatty, (B) breast has scattered areas of fibroglandular density, (C) breast is heterogeneously dense, and (D) breast is extremely dense] were created with a voxel size of 0.125 mm. Of these NBPs, 44 have shapes compatible with the patient’s position during a 3D OAT scan and the others have hemispherical shapes compatible to the use of breast cups to stabilize the breast during imaging.^3^^,^^6^ Three target wavelengths of 757, 800 (the isosbestic point of deoxy- and oxy-hemoglobin), and 850 nm were selected. Four lesions of different sizes, each smaller than 10 mm in diameter and composed solely of a VTC region, were inserted into each of 80 NBPs (40 natural-shaped and 40 hemispherical-shaped NBPs). Two lesions, one with a necrotic core and PA region and the other without, were inserted into each of the remaining four natural-shaped NBPs [Fig. 4].

The generation of anatomical structures of the breast using the VICTRE tool with the modifications in terms of the blood vessel trees described in Sec. 3.1.2 took approximately 100 to 310 mins using a 16-core Intel Xeon Gold 6130 CPU and 256 GB of memory. The computation time varied depending on the volume of the phantoms. Note that breast sizes, shapes, and structures varied among NBPs [see Fig. 4]. The computation time to generate and insert the blood vasculature under the skin layer and the lesions was 1 to 6 mins (depending on the phantom volume), and the assignment of functional, optical, and acoustic properties took approximately 40, 15, and 2 mins, respectively, using the same machine.

**Fig. 5:**
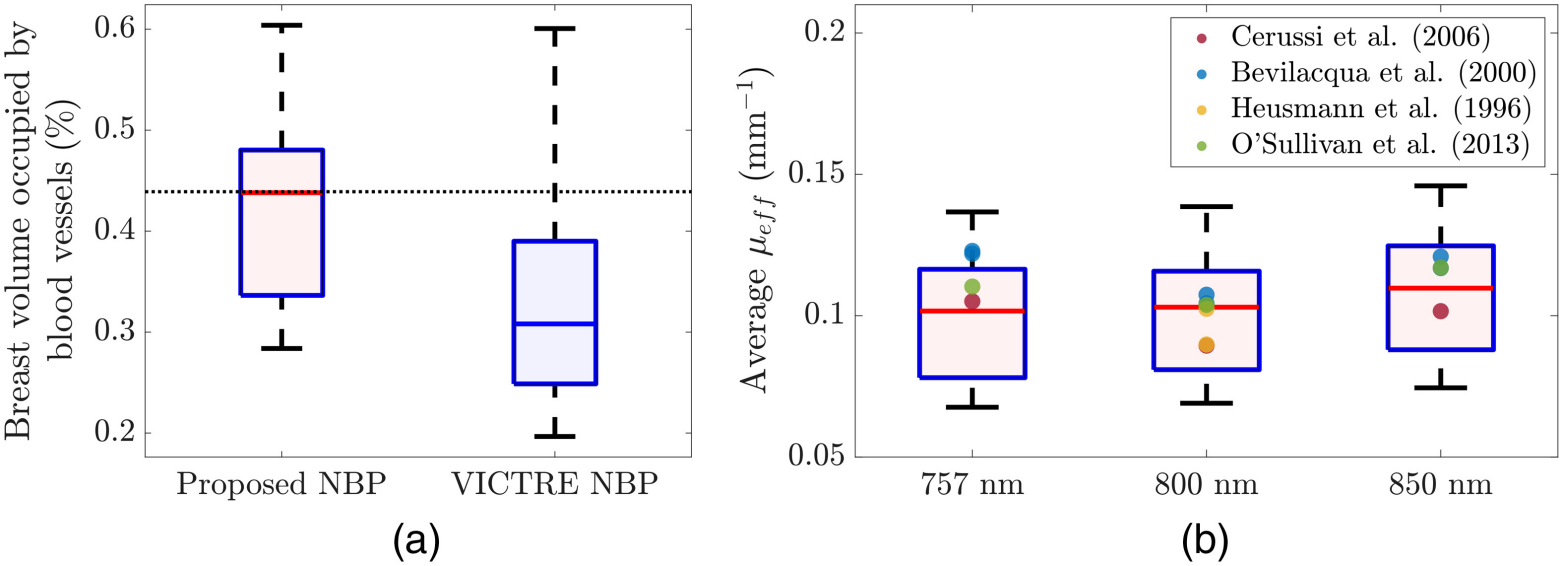
Box plots of (a) breast volume percentages occupied by blood vessels in which 40 proposed NBPs (left) and 40 unmodified VICTRE NBPs (right) are compared and (b) spatially averaged effective optical attenuation coefficients $\mu_{\mathrm{eff}}$ estimated from 40 proposed NBPs at wavelengths of 757, 800, and 850 nm (left to right). The used NBPs have hemispherical shapes with radii stochastically sampled from the probability distribution ($a_{1t}$) in Table 2. In panel (a), the black dotted horizontal line indicates the estimated mean of four clinical OAT images acquired by TomoWave Laboratories (Houston, Texas, United States) using LOUISA-3D^3^ at the MD Anderson Cancer Center. In panel (b), the reference values were calculated from the measurements in Refs. 51 and 85–87.

Figure 5(a) presents box plots of the breast volume percentages occupied by blood vessels in which 40 proposed NBPs and 40 unmodified VICTRE NBPs are compared. Figure 5(b) shows box plots of the spatially averaged effective optical attenuation $\mu_{\mathrm{eff}}$ estimated from 40 proposed NBPs at the three wavelengths via the Beer–Lambert law-based estimation method in Ref. 33.

As shown in Fig. 5(a), the reference value (0.439%) of the breast volume percentage occupied by blood vessels, i.e., an estimated mean of the clinical OAT images (a dotted horizontal line), is within the interquartile range of the proposed NBPs (0.336% to 0.48%, median of 0.438%), but it is not within the interquartile range of the VICTRE NBPs (0.249% to 0.39%, median of 0.31%). In Fig. 5(b), the $\mu_{\mathrm{eff}}$ estimates of female breasts reported in Ref. 51 and 85–87 all fall within the interquartile range of the proposed NBPs for light excitation wavelengths of 800 and 850 nm. For a light wavelength of 757 nm, the two $\mu_{\mathrm{eff}}$ estimates from Ref. 51 are slightly out of the interquartile range of the proposed NBPs (the 84th and 85th percentiles). Once more data on the functional and optical properties of breasts, specifically the $f_{w}$ and $\mu_{a}$ values of breast fat and glandular tissues for different breast types, become available and are incorporated into NBPs, the $\mu_{\mathrm{eff}}$ values are expected to better match the reference values found in the existing literature.

### 4.2 Example OAT Images from Simulated Data

**Fig. 6:**
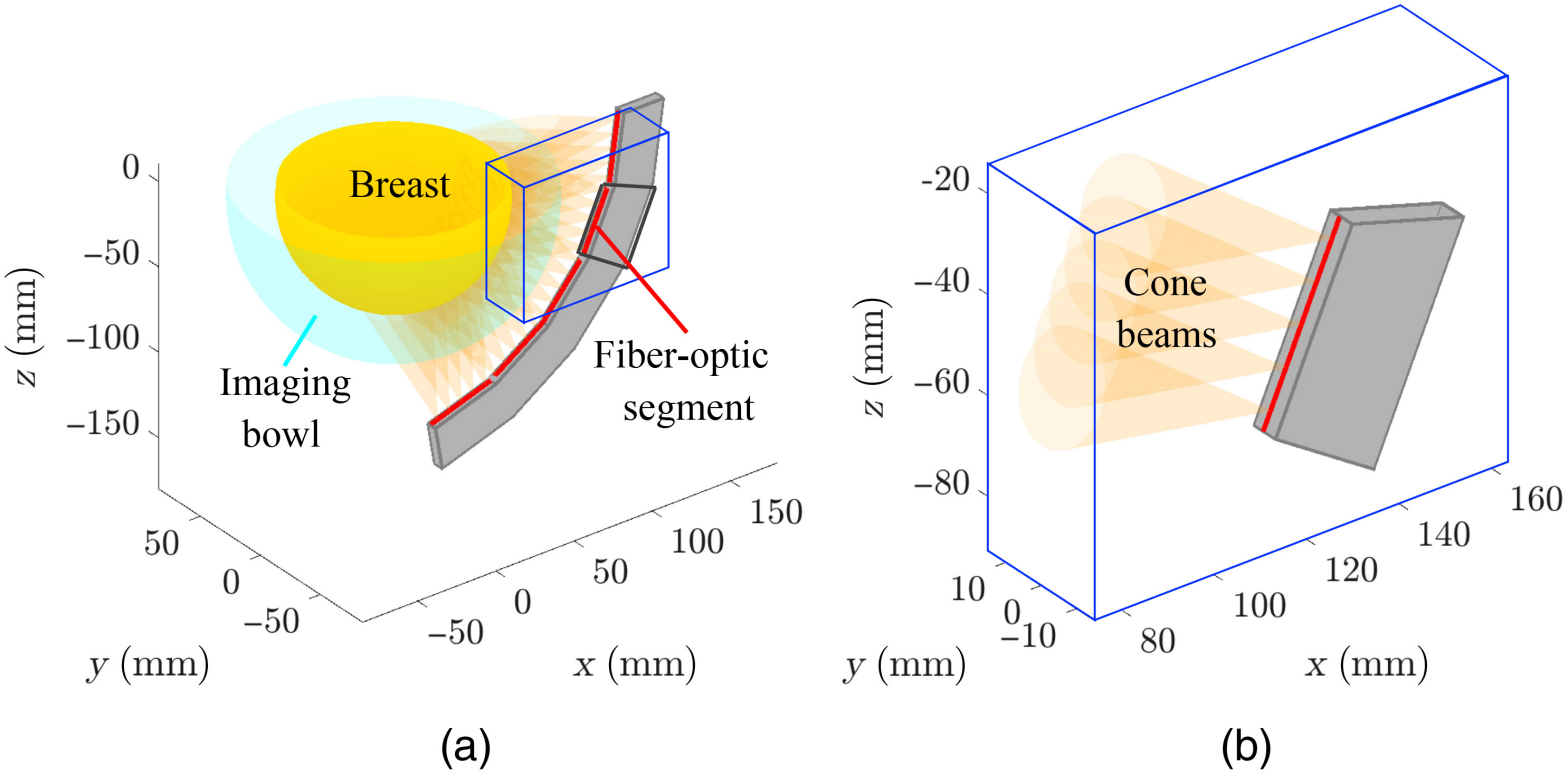
Virtual light delivery system: (a) five linear fiber-optic segments (red lines) are attached on the surface of an arc-shaped illuminator (radius of 145 mm, central angle of 80 deg), and (b) five cone beams (half-angle of 12.5 deg) are emitted from the locations uniformly distributed on each linear fiber-optic segment. Panels (a) and (b) show light delivery at an illumination view. The total number of illuminator arcs is 20, so 500 cone beams illuminate the breast.

OAT images that depict the induced pressure distributions were reconstructed from simulated measurement data corresponding to the considered NBPs. The light delivery subsystem of the virtual OAT imaging system consisted of 20 arc-shaped illuminators uniformly distributed along the azimuthal angle. Each illuminator arc had a radius of 145 mm and was modeled using 25 cone beam sources as illustrated in Fig. 6. The acoustic detection subsystem consisted of 51,472 transducer elements uniformly distributed on a hemispherical aperture with a scanning radius of 85 mm. Each virtual transducer element recorded 3,720 time samples of pressure data at a sampling frequency of 20 MHz. Additional details on the virtual OAT imaging system are presented in Appendix C.

**Fig. 7:**
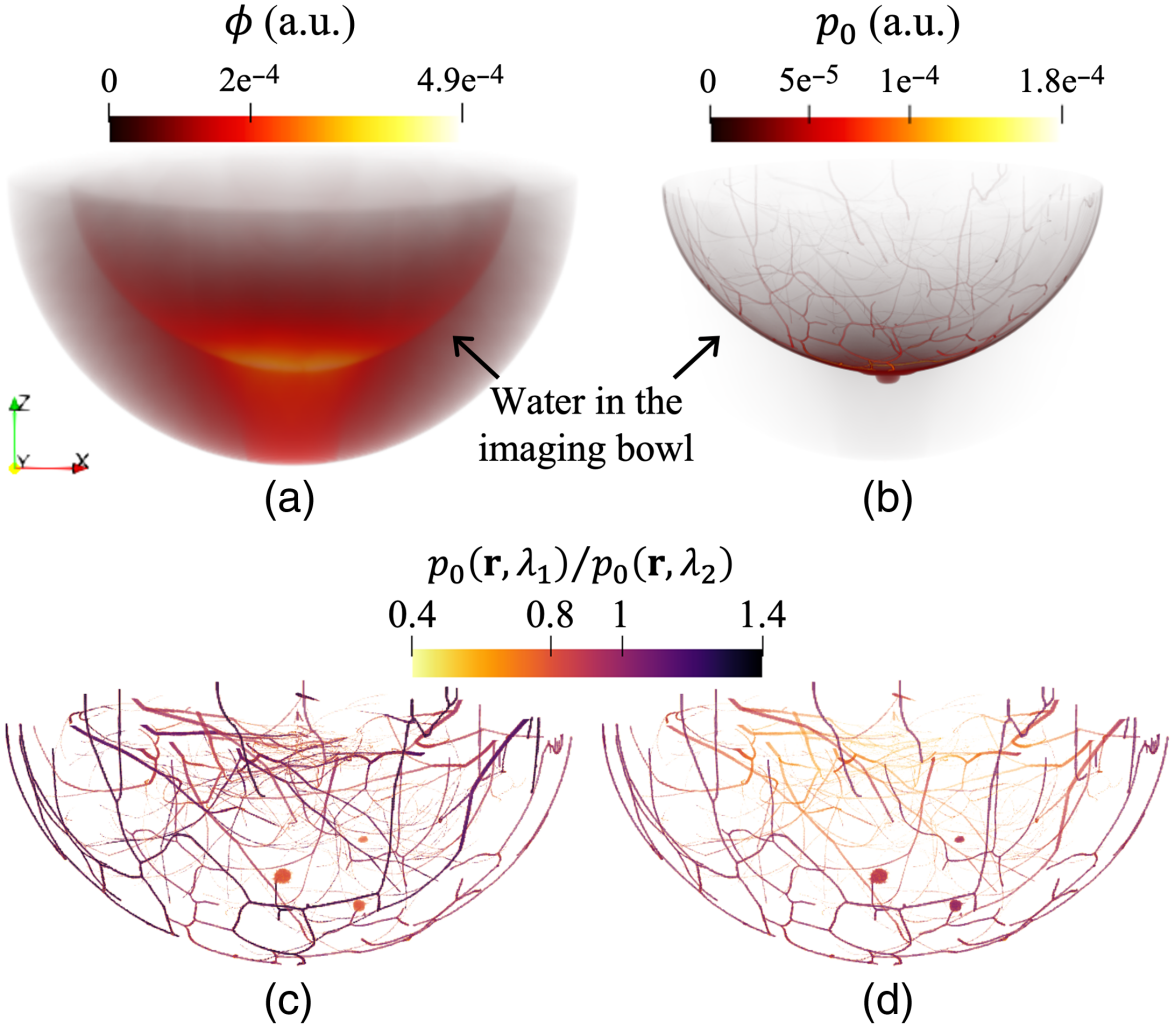
Simulated distributions of optical fluence ϕ and initial pressure $p_{0}$: (a) ϕ and (b) $p_{0}$ of the breast in the water-filled imaging bowl at a wavelength of 800 nm, and the $p_{0}$ ratio of the breast blood vasculature and lesions between wavelengths (c) of 800 and 757 nm and (d) of 850 and 800 nm. These are from a type C breast. Paraview^40^ was used for volume rendering, and color maps were adjusted for better visibility.

OAT data were virtually acquired via multiphysics simulation of the photoacoustic effect and subsequent wave propagation. First, the simulation of photon transport in biological tissues was conducted using the GPU-accelerated MCX version 1.9.0^23^^,^^24^ to compute the induced initial pressure distribution at the three optical wavelengths of interest (757, 800, and 850 nm). Figures 7(a) and 7(b) illustrate the simulated distributions of optical fluence ϕ and true initial pressure $p_{0}$, and Figs. 7(c) and 7(d) show the $p_{0}$ ratios of the breast blood vasculature and lesions between wavelength pairs of 800 and 757 nm and of 850 and 800 nm, respectively.

**Fig. 8:**
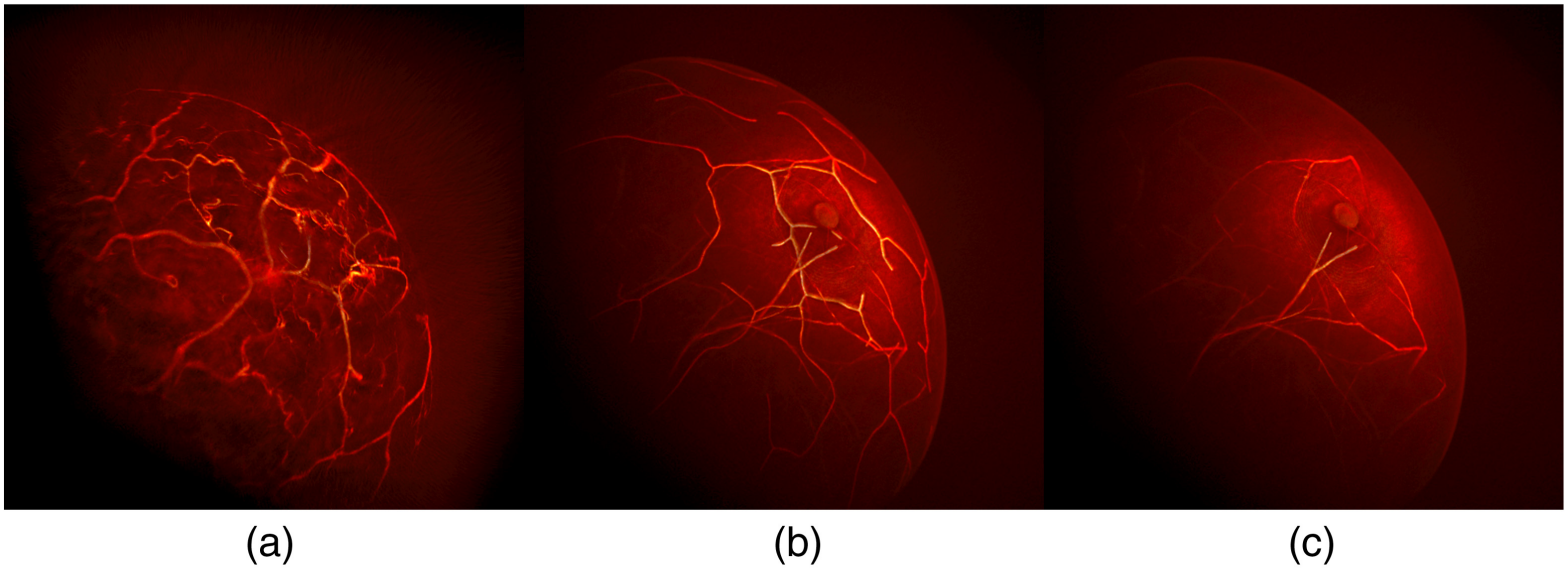
Visual comparison of 3D OAT breast images reconstructed from (a) clinical measurement data and simulated measurement data produced using (b) the proposed NBP and (c) the unmodified VICTRE NBP. In panel (a), the clinical data were acquired by TomoWave Laboratories using LOUISA-3D.^3^ In the unmodified VICTRE NBP, tissue properties were assigned to each tissue type in a piecewise constant manner. All three images were reconstructed using the FBP method.^42^ The images were visualized using Paraview’s volume rendering technique, which accumulates intensities based on the selected color and opacity maps.^40^

Next, wave propagation in acoustically heterogeneous media was simulated using the GPU-accelerated k-Wave toolbox version 1.3,^25^ and pressure traces measured by the virtual transducer elements were recorded. Finally, measurement noise was modeled as independent and identically distributed Gaussian noise with zero mean and a standard deviation equal to 1% of the maximum optoacoustic signal strength of the entire ensemble for all three wavelengths, as was empirically determined based on the *in vivo* breast OAT data.^3^^,^^33^ Using the simulated noisy acoustic measurements, the images, i.e., $p_{0}$ estimates, were reconstructed employing the FBP method with a voxel size of 0.25 mm. Figure 8 presents a visual comparison of 3D OAT images reconstructed from clinical measurement data and simulated measurement data produced using the proposed NBP and the unmodified VICTRE NBP. The OAT image generated using the proposed NBPs [Fig. 8(b)] plausibly resembles the 3D clinical OAT image [Fig. 8(a)], compared with the unmodified VICTRE NBP [Fig. 8(c)].

## 5 Case Study: Acoustic Heterogeneity in Image Reconstruction

To demonstrate the usefulness of the proposed framework in virtual OAT studies, a case study was conducted to explore the impact of acoustic heterogeneity in OAT images of the breast. One phantom for each breast type (A, B, C, or D), four in total, was used for this case study. Acoustic measurements were simulated via virtual OAT data acquisition based on the target imaging system described in Sec. 4.2.

**Fig. 9:**
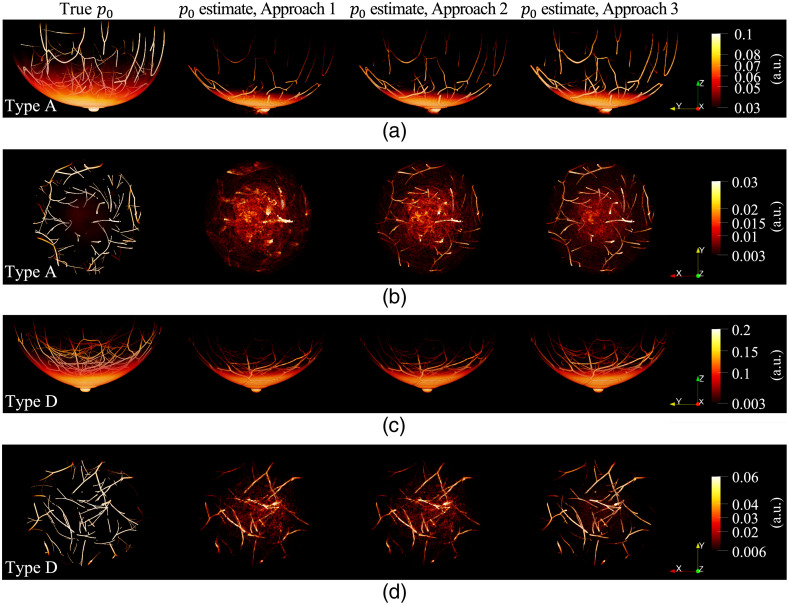
True distributions of initial pressure $p_{0}$ at a wavelength of 800 nm (first column) and $p_{0}$ estimates reconstructed assuming homogeneous acoustic properties of water (second column), two sound speeds for water and breast tissue regions each and homogeneous density and acoustic attenuation properties of water (third column), and true distributions of acoustic properties (fourth column). The whole breast regions are presented from a side view in panels (a) and (c), and the regions at depths between 10 and 20 mm from the breast surface are illustrated from a front view in panels (b) and (d). The images in panels (a) and (b) are from a type A breast, and those in panels (c) and (d) are from a type D breast. Paraview^40^ was used for volume rendering, and color maps were adjusted for better visibility.

To explore the impact of acoustic heterogeneity in OAT images of the breast, the images, i.e., $p_{0}$ estimates, were reconstructed from the noisy acoustic measurements simulated in Sec. 4.2 with a voxel size of 0.25 mm. Three different approaches were used, each with the following assumptions: homogeneous acoustic properties of water in Table 7 for the entire computation domain (Approach 1); two sound speed values for water ($c_{\text{water}}$) and breast tissue ($c_{\text{breast}}$) each, and homogeneous density and acoustic attenuation properties of water for the whole domain (Approach 2); and the true distributions of acoustic properties (Approach 3). For image reconstruction, the LSQR^88^ algorithm was implemented^89^ to compute a least-squares estimate of $p_{0}$. As the stopping rule, the iteration was terminated when the squared error between the true $p_{0}$ and estimated $p_{0}$ started to increase to avoid overfitting the noisy data. The goal of this case study was not to illustrate a practical image reconstruction algorithm but to investigate the effect of acoustic heterogeneity on image quality. Thus, a stylized stopping criterion, which requires knowledge of the true object, was purposely chosen to isolate the effect of acoustic heterogeneity from other sources of error or bias in OAT images, such as the design of an appropriate regularization functional. Figure 9 shows the true $p_{0}$ (first column) and the $p_{0}$ estimates (second to fourth columns) of Approaches 1 to 3, respectively. To tune $c_{\text{breast}}$ in Approach 2, sound speed values between 1.45 and 1.54  mm/μs were swept with a step size of 0.005  mm/μs, and the selected values for each breast type are presented in Table 8.

**Table 8:** Average relative squared error (RSE) and structural similarity index (SSIM).

Breast type	$c_{\text{breast}}$ (mm/μs)	RSE			SSIM		
		Approach 1	Approach 2	Approach 3	Approach 1	Approach 2	Approach 3
A	1.47	0.4605	0.4550	0.3637	0.9808	0.9827	0.9838
B	1.475	0.4296	0.4275	0.3256	0.9790	0.9801	0.9833
C	1.495	0.4291	0.4146	0.3238	0.9798	0.9807	0.9834
D	1.53	0.3552	0.3464	0.2699	0.9801	0.9801	0.9835

$c_{\text{breast}}$: breast sound speed used in Approach 2.

The relative squared error $\mathrm{RSE}={\left\|x_{\text{true}}-x_{\mathrm{est}}\right\|}_{2}^{2}/{\left\|x_{\text{true}}\right\|}_{2}^{2}$ and structural similarity index measure (SSIM)^90^ between the true $p_{0}$ ($x_{\text{true}}$) and the reconstructed image ($x_{\mathrm{est}}$) were calculated to quantify the accuracy of the reconstructed images. Table 8 provides the average RSE and SSIM calculated from the data of breast types A to D for all three wavelengths. The RSE and SSIM improved for all breast types when acoustic heterogeneity was accounted for in the image reconstruction. As shown in Fig. 9, the mitigation of clutter artifacts using the two-sound speed model (Approach 2) with respect to the homogeneous model (Approach 1) was more significant in the type A breast (almost entirely fatty breast) than the type D breast (extremely dense breast). Here, clutter refers to background artifacts caused by uncompensated scattering or refraction effects. For the fatty type breast, the sound speed mismatch between fatty tissue and water is the main cause of clutter; therefore, the two-sound speed model (Approach 2) can effectively mitigate such artifacts. However, for denser type breasts, glandular tissue has a sound speed similar to water, and acoustic impedance heterogeneity across different breast tissues is the primary cause of clutter; thus the two-sound speed model is less effective.

## 6 Conclusion

In this work, a framework to generate realistic 3D NBPs that can be used in large-scale VITs of OAT breast imaging was established. For the first time, anatomically, physiologically, and optoacoustically realistic 3D NBPs and NLPs in varying sizes and shapes could be produced using the proposed framework. This was achieved by extending VICTRE tools to OAT imaging with substantial modifications. Because the proposed framework was implemented in a modular form, it facilitates further customization of the NBPs. Users can replace the relatively simple lesion model applied in this study with more biologically realistic models of tumor growth and metabolism. For example, heterogeneous distributions of multiple necrotic regions and blood vessels proliferating toward the lesion could be included. Future studies may also include explicit geometric modeling of vascular growth associated with tumors, functional and optical modeling of a specific type of breast cancer, analysis of lesion detectability as a function of their size and depth, and mechanical deformations of the breast to simulate slight breast compression in the existing OAT system. In summary, the produced NBPs and NLPs enhance the authenticity of virtual OAT studies, and the proposed framework can be widely employed for the investigation and development of advanced image reconstruction methods and machine learning-based methods, as well as the objective evaluation and optimization of the OAT breast imaging systems. Code packages of the proposed tools and 84 datasets that were made publicly available will enable researchers to immediately benefit from this work.

## 7 Appendix A: Generation of Blood Vasculature under the Skin Layer

Blood vasculature under the skin layer [Figs. 2(a) and 2(d)] was stochastically generated visually similar to the blood vessels observed in the clinical OAT images^3^^–^^6^^,^^33^ via relatively computationally inexpensive image processing methods (random sampling, Gaussian blurring, Otsu’s thresholding,^44^ and skeletonization) that are commonly used.

In Algorithm 1, the inputs $N_{x}$, $N_{y}$, and $N_{z}$ are the sizes of the anatomical NBP volume along the x-, y-, and z-axes; inputs $\sigma_{1}$ and $\sigma_{2}$ of Gaussian filters control the distance between blood vessel branches in the vessel tree; and input $\sigma_{3}$ determines the thickness of blood vessels under the skin layer. Based on the breast volume percentage occupied by blood vessels estimated from the clinical OAT images, the values of $\sigma_{1}$, $\sigma_{2}$, and $\sigma_{3}$ were set to 3.875, 6.125, and 0.219 mm (corresponding to the vessel diameter of 0.75 mm), respectively. The erosion in step (7) of Algorithm 1 shrinks bright regions in the breast mask without skin, i.e., voxels under the breast skin. Thus, the degree of erosion determines the distance between the inserted blood vasculature and the skin. For example, three erosion iterations at a voxel size of 0.125 mm yield the blood vessels at a depth of 0.375 mm under the skin. The proposed method does not produce a specific number and volume of arteries and veins under the skin layer. Instead, it stochastically creates visually plausible vascular structures employing computationally efficient methods, so the number and volume of the blood vessel segments produced in step (11) of Algorithm 1 are not always even for arteries and veins. The blood vessel segments were assigned to the artery mask and vein mask alternately, depending on the segment locations, in step (12) of Algorithm 1.

**Algorithm 1:** Blood vasculature under the skin layer

**Input:** breast mask without skin, $N_{x}$, $N_{y}$, $N_{z}$, $\sigma_{1}$, $\sigma_{2}$, $\sigma_{3}$
**Output:** artery mask, vein mask
1: Obtain a $N_{y}\times N_{z}$ random sample matrix from a uniform distribution
2: Obtain two blurred matrices via Gaussian filtering with $\sigma_{1}$ and $\sigma_{2}$ to the result of step (1)
3: Calculate the difference between the results of step (2)
4: Binarize the result of step (3) by Otsu’s thresholding
5: Skeletonize the result of step (4)
6: Obtain a $N_{x}\times N_{y}\times N_{z}$ matrix that contains $N_{x}$ copies of the result of step (5) in the first dimension of 3D
7: Perform bit-wise exclusive or operation (XOR) between breast mask without skin and that after erosion for 0.375 mm
8: Perform XOR of the results of steps (6) and (7)
9: Skeletonize the result of step (8)
10: Blur the result of step (9) by Gaussian filtering with $\sigma_{3}$
11: Binarize the result of step (10) by Otsu’s thresholding
12: Obtain artery mask and vein mask by labeling the segments in the result of step (11) with either arteries or veins, respectively
13: **return** artery mask, vein mask

The produced blood vasculature was embedded into the anatomical NBPs by assigning the artery label to the artery mask region and the vein label to the vein mask region in the anatomical NBPs [Figs. 2(a) and 2(d)].

## 8 Appendix B: Modeling Distributions of Oxygen Saturation and Blood Volume Fraction

To model a spatially varying distribution of oxygen saturation s(r) at each spatial coordinate r within the phantom, the following diffusion-reaction PDE^81^ was solved:

$$-\mu \Delta s(r)+\underset{i\in T_{s}}{\sum }\chi_{i}(r)(s(r)-{\overset{¯}{s}}_{i})=0.$$

Here, μ>0 is a numerical parameter controlling the smoothness of the oxygen saturation s(r); Δ is the Laplace operator; $T_{s}=\{$ skin, artery, vein, lesion } is a set of tissues of interest; $\chi_{i}(r)$ and ${\overset{¯}{s}}_{i}$ are the indicator function and the target value of oxygen saturation, respectively, (randomly sampled according to the tissue-specific probability distributions in Table 5) assigned to the tissue i ($i\in T_{s}$).

Tumor angiogenesis was modeled in the blood volume fraction distribution $f_{b}(r)$ at a spatial coordinate r within the phantom by solving the following diffusion-reaction PDE:

$$-\mu \Delta f_{b}(r)+\underset{i\in T_{b}}{\sum }\chi_{i}(r)(f_{b}(r)-{\overset{¯}{f_{b}}}_{i})=0,$$

where $T_{b}=\{$ VTC, necrotic core, fat/ligament/TDLU/duct, glandular, artery, and vein } is a set of tissues of interest and ${\bar{f_{b}}}_{i}$ is the target value of the tissue blood volume fraction sampled from the predefined probability distributions in Table 5 for the tissue i ($i\in T_{b}$).

The PDEs above were solved using a linear finite element on an unstructured tetrahedral mesh that is fitted to tissue interfaces. Unstructured meshes were generated using the pygalmesh software,^91^^,^^92^ and the PDE was solved using the parallel finite element library FEniCS.^82^

## 9 Appendix C: Details of Virtual OAT Imaging System and Data Acquisition

The light delivery mechanism and the measurement geometry of the target virtual OAT imaging system were chosen to emulate one of the existing OAT systems for breast imaging, LOUISA-3D^3^^,^^33^ developed by TomoWave Laboratories (Houston, Texas, United States). The detailed illumination geometry of the virtual light delivery system using 20 arc-shaped illuminators [see Fig. 6] is described in Table 9.

**Table 9:** Light delivery geometry of arc-shaped illuminators.

Parameter	Description
Illuminator radius and central angle	145 mm, 80 deg
Number of illuminators	20
Number of fiber-optic segments	5 (linear shape) per illuminator
Light source	500 cone beams (5 per fiber segment ×5 segments per view ×20 illuminator arcs) with a half-angle of 12.5 deg
Light direction	Perpendicular to the linear fiber segment toward the chest wall center

For the virtual OAT data acquisition, idealized point-like transducers, uniformly distributed on the arc-shaped optoacoustic probe specified in Table 10, were assumed; the probe collects a total of 3,720 time samples of pressure data at a sampling frequency of 20 MHz. For the simulation of OAT data acquisition, the k-Wave GPU code was used as the acoustic wave propagation simulator. With the assumption of idealized point-like transducers, the possible option to define transducer locations in the simulation supported by the k-Wave GPU code is a binary matrix, where the value at the corresponding transducer locations is “1” and at the others is “0.” Therefore, 51,472 transducer locations were defined as a target measurement geometry, excluding the overlaps due to the discretization of Cartesian coordinates into the binary matrix with the given voxel size.

**Table 10:** Measurement geometry of the arc-shaped optoacoustic probe.

Parameter	Description
Number of tomographic views	480
Scanning radius	85 mm
Probe central angle	80 deg
Number of transducer elements	108 per probe

Acoustic OAT measurements were virtually acquired through the simulation of photon transport in breast tissues, calculation of initial pressure distribution $p_{0}$, and simulation of acoustic wave propagation. In the simulation of the photon transport using the GPU-accelerated MCX, the light source and direction parameters were set according to the target system design [Fig. 6 and Table 9], and the simulation domain size was set to 340×340×170  voxels with a spatial step size of 0.5 mm. Among the optical NBPs employed as inputs of the simulation, the distributions $\mu_{a}$ and $\mu_{s}$ for wavelengths of 757, 800, and 850 nm were downsampled using linear interpolation (from a voxel size of 0.125 to 0.5 mm), and constant values g and n were averaged within breast tissues. With these inputs, the distributions of optical fluence ϕ were simulated using $10^{8}$ photons per beam for a time duration of 5 ns.

The true $p_{0}=\Gamma \mu_{a}\phi$ at a voxel size of 0.25 mm [Fig. 7(b)] was calculated via elementwise multiplication of $\mu_{a}$ and the simulated ϕ, assuming a constant Grüneisen parameter Γ=1, as is commonly done for soft tissues.^16^^,^^33^ The $\mu_{a}$ and the simulated ϕ were downsampled (from a voxel size of 0.125 to 0.25 mm) and upsampled (from a voxel size of 0.5 to 0.25 mm) via linear interpolation, respectively. The MCX simulation at a coarser grid accompanied by the upsampling not only is equivalent to smoothing the $p_{0}$ in the acoustic wave propagation simulation to reduce the amplitudes of the high spatial frequency components but also significantly reduces the computation time of the MCX simulation.

In the simulation of acoustic wave propagation using the k-Wave toolbox and its GPU code,^25^ the measurement geometry was configured based on the target system design [Table 10] as explained above. The voxel size was set to the smallest possible, i.e., 0.25 mm, given the measurement geometry and the use of an NVIDIA Tesla V100 GPU with 32 GB memory. The simulation domain size was set to 700×700×350  voxels. The acoustic NBPs c, ρ, and $\alpha_{0}$ employed to simulate acoustic measurements were downsampled using linear interpolation (from a voxel size of 0.125 to 0.25 mm). An anisotropic absorbing boundary layer, known as a perfectly matched layer, was set to be outside the simulation domain to prevent undesired acoustic reflection at the boundaries. A pressure wavefield generated by the simulated $p_{0}$ was virtually propagated based on the acoustic NBPs and measured by the virtual transducer elements in the target imaging system.

## Data, Materials, and Code Availability

Eighty-four datasets (21 per breast type) have been publicly released on the Harvard Dataverse.^28^^,^^29^^,^^30^ Each dataset includes a natural-^28^ or hemispherical-shaped anatomical NBP^29^ with four NLPs inserted, which are composed solely of a VTC region, or a natural-shaped anatomical NBP^30^ with two NLPs inserted, one with a necrotic core and PA region and the other without, as described in Sec. 3.1; distributions of functional ($c_{\mathrm{tHb},\text{blood}}$, s, $f_{b}$, $f_{w}$, $f_{f}$, and $f_{m}$), optical ($\mu_{a}$, $\mu_{s}$, g, and n), and acoustic properties (c, ρ, $\alpha_{0}$, and y), as described in Sec. 3.2; and simulated multi-wavelength optical fluence, initial pressure, and OAT measurements, as explained in Sec. 5. Our modified version of the VICTRE tool code package and the Python library SOA-NBP implementing the methods presented in this work are available under CC0 and GPLv3 from Refs. 26 and 27, respectively. Information on how to contribute to our toolkit and developer guidelines can be found in Ref. 27.
